# Supplementary material for: Environmental Enrichment Engages Vesicular Zinc Signaling to Enhance Hippocampal Neurogenesis
Source: Cells. 2023 Mar 13;12(6):883. doi: 10.3390/cells12060883 (PMC10046929; doi:10.3390/cells12060883)
Supplement: Supplementary file 1 [file cells-12-00883-s001.zip › cells-2242321-supplementary.pdf]

SUPPLEMENTAL TABLES

Supplementary Table S1. Two-way ANOVA statistical results (effects of genotype and housing condition).

**Cell Proliferation**	*df*	*F*	*p*
Genotype	1, 20	12.51	**.002***
Housing	1, 20	15.18	**< .001***
Genotype × Housing	1, 20	12.31	**.002***
**Cell Survival**	*df*	*F*	*p*
Genotype	1, 19	22.54	**< .001***
Housing	1, 19	31.02	**< .001***
Genotype × Housing	1, 19	17.71	**< .001***
**Neuronal Differentiation**	*df*	*F*	*p*
Genotype	1, 19	.013	.910
Housing	1, 19	92.45	**< .001***
Genotype × Housing	1, 19	0.29	.596
**Cell Death**	*df*	*F*	*p*
Genotype	1, 19	48.97	**< .001***
Housing	1, 19	71.12	**< .001***
Genotype × Housing	1, 19	30.94	**< .001***
**Granule Cell Layer Volume**	*df*	*F*	*p*
Genotype	1, 19	22.78	**< .001***
Housing	1, 19	17.84	**< .001***
Genotype × Housing	1, 19	19.72	**< .001***
**Spatial Object Recognition**	*df*	*F*	*p*
Genotype	1, 35	6.96	**.012***
Housing	1, 35	8.72	**.006***
Genotype × Housing	1, 35	7.12	**.011***
**BDNF**	*df*	*F*	*p*
Genotype	1, 28	7.77	**.009***
Housing	1, 28	9.08	**.005***
Genotype × Housing	1, 28	5.28	**.029***

Note. df = degrees of freedom. ***p* < .05**.

Supplementary Table S2. Three-way repeated measures ANOVA statistical results (effects of genotype, housing condition, and day) of escape latency in the Morris Water Task.

	df	*F*	*p*
Day	2, 70	29.16	**< .001***
Genotype	1, 35	1.99	.167
Housing Condition	1, 35	6.23	**.017***
Day × Genotype	2, 70	0.93	.400
Day × Housing Condition	2, 70	1.98	.146
Genotype × Housing Condition	1, 35	12.18	**.001***
Day × Genotype × Housing Condition	2, 70	0.28	.754

Note. df = degrees of freedom. ***p* < .05**.

Supplementary Table S3. Levene's test of homogeneity of variance for two-way ANOVAs (effects of genotype and housing condition).

	df	Statistic	*p*
Cell Proliferation	3, 20	1.03	.403
Cell Survival	3, 19	1.54	.236
Neuronal Differentiation	3, 19	2.55	.086
Cell Death	3, 19	0.31	.821
Granule Cell Layer Volume	3, 19	0.39	.764
Spatial Object Recognition	3, 35	0.44	.728
BDNF	3, 28	0.06	.981

Note. Results are based on mean. *df* = degrees of freedom. ***p* < .05**.

Supplementary Table S4. Mauchly's test of sphericity and Levene's test of homogeneity of variance for three-way repeated measures ANOVA (effects of genotype, housing condition, and day).

**Mauchly's Test**	*df*	Mauchly's *W*	*p*
Day	2, 70	.98	.661
**Levene's Test**	*df*	Statistic	*p*
Morris Water Task – Day 1	3, 35	1.38	.264
Morris Water Task – Day 2	3, 35	1.21	.322
Morris Water Task – Day 3	3, 35	1.79	.166

Note. Results are based on mean. *df* = degrees of freedom. **$^*p < .05$**.

Supplementary Table S5. Levene's test of homogeneity of variance for independent samples t-tests (effect of treatment) separated by genotype.

**WT**	Statistic	Significance	*t*	*df*	*p*
Equal variances assumed	0.14	.710	2.33	14	**.018***
Equal variances not assumed			2.33	13.70	.018*
**ZnT3 KO**	Statistic	Significance	*t*	*df*	*p*
Equal variances assumed	3.52	.088	2.19	11	**.025***
Equal variances not assumed			2.28	9.92	.023*

Note. Results are based on mean. *df* = degrees of freedom. ***p* < .05**. When the assumption of homogeneity of variances was not met (**significance < .05**), the *t*, *df*, and *p* values from the "equal variances not assumed row" was used.

Supplementary Table S6. Additional statistical data for all statistical analyses. Statistics are reported as mean ± standard error of the mean.

	WT-SH	WT-EE	ZnT3 KO-SH	ZnT3 KO-EE
Cell Proliferation	4016.00 ± 105.48 (*n* = 6)	5067.00 ± 182.26 (*n* = 6)	4012.00 ± 139.67 (*n* = 6)	4067.00 ± 129.45 (*n* = 6)
Cell Survival	852.00 ± 43.90 (*n* = 6)	1722.00 ± 113.73 (*n* = 6)	804.00 ± 60.14 (*n* = 6)	925.20 ± 123.54 (*n* = 5)
Neuronal Differentiation (% of BrdU-positive cells co-labelled with NeuN)	65.34 ± 4.42 (*n* = 6)	92.36 ± 1.23 (*n* = 6)	63.40 ± 3.16 (*n* = 6)	93.63 ± 1.25 (*n* = 5)
Cell Death	488.00 ± 10.70 (*n* = 6)	293.00 ± 12.81 (*n* = 6)	508.00 ± 12.17 (*n* = 6)	468.00 ± 20.35 (*n* = 5)
Granule Cell Layer Volume (mm^3)	1.02 ± 0.03 (*n* = 6)	1.33 ± 0.04 (*n* = 6)	1.00 ± 0.04 (*n* = 6)	0.99 ± 0.05 (*n* = 5)
Spatial Object Recognition (Investigation ratio; %)	49.83 ± 2.61 (*n* = 10)	66.08 ± 3.30 (*n* = 9)	49.94 ± 2.51 (*n* = 10)	50.74 ± 3.13 (*n* = 10)
Morris Water Task – Day 1 (Escape latency; s)	40.11 ± 3.27 (*n* = 10)	29.21 ± 4.23 (*n* = 9)	37.65 ± 3.38 (*n* = 10)	44.65 ± 2.69 (*n* = 10)
Morris Water Task – Day 2 (Escape latency; s)	36.91 ± 2.90 (*n* = 10)	17.04 ± 2.73 (*n* = 9)	30.96 ± 3.13 (*n* = 10)	29.05 ± 4.09 (*n* = 10)
Morris Water Task – Day 3 (Escape latency; s)	25.07 ± 4.81 (*n* = 10)	14.93 ± 4.85 (*n* = 9)	19.28 ± 2.04 (*n* = 10)	20.98 ± 3.29 (*n* = 10)
BDNF Levels (pg/mg total protein)	314.66 ± 25.56 (*n* = 8)	455.86 ± 27.06 (*n* = 8)	301.66 ± 26.33 (*n* = 7)	320.67 ± 26.44 (*n* = 9)
	**WT**	**WT-Zn**	**ZnT3 KO**	**ZnT3 KO-Zn**
Percent Change in BDNF (from control; %)	100.00 ± 7.76 (*n* = 8)	125.93 ± 9.01 (*n* = 8)	100.00 ± 4.58 (*n* = 6)	119.41 ± 7.16 (*n* = 7)
